# Determination of Dopamine in the Presence of Ascorbic Acid by Nafion and Single-Walled Carbon Nanotube Film Modified on Carbon Fiber Microelectrode

**DOI:** 10.3390/s8116924

**Published:** 2008-11-04

**Authors:** Haesang Jeong, Seungwon Jeon

**Affiliations:** Department of Chemistry and Institute of Basic Science, Chonnam National University, Gwangju, 500-757, Korea; E-Mail: haesang70@hanafos.com (H. Jeong)

**Keywords:** Single-walled carbon nanotubes, Nafion, Carbon fiber microelectrode, Dopamine, Ascorbic acid, Electrocatalytic oxidation

## Abstract

Carbon fiber microelectrode (CFME) modified by Nafion and single-walled carbon nanotubes (SWNTs) was studied by voltammetric methods in phosphate buffer saline (PBS) solution at pH 7.4. The Nafion-SWNTs/CFME modified microelectrode exhibited strongly enhanced voltammetric sensitivity and selectivity towards dopamine (DA) determination in the presence of ascorbic acid (AA). Nafion-SWNTs film accelerated the electron transfer reaction of DA, but Nafion film as a negatively charged polymer restrained the electrochemical response of AA. Voltammetric techniques separated the anodic peaks of DA and AA, and the interference from AA was effectively excluded from DA determination. Linear calibration plots were obtained in the DA concentration range of 10 nM - 10 μM and the detection limit of the anodic current was determined to be 5 nM at a signal-to-noise ratio of 3. The study results demonstrate that DA can be determined without any interference from AA at the modified microelectrode, thereby increasing the sensitivity, selectivity, and reproducibility and stability.

## 1. Introduction

Dopamine (DA) has long been of interest to neuroscientists and chemists because of its role as a very important neurotransmitter in mammalian central nervous systems and the fact that low levels of DA concentration bring about Parkinson's disease [1]. A major problem for the electrochemical detection of DA in blood samples is the presence of many interfering compounds. In particular, ascorbic acid (AA), which has a similar oxidation potential and is usually present *in vivo* at concentrations 10^2^ to 10^3^ times higher than those of DA. A conventional way to solve this problem is to coat the electrode surface with an anionic film to protect the surface from interference.

Since their discovery carbon nanotubes (CNTs) have attracted much research interest as novel materials with excellent electrical conductivity, mechanical strength, chemical stability and flexibility properties [2]. They present several forms, including single- (SWNTs), double- (DWNTs), and multi-walled carbon nanotubes (MWNTs). SWNTs are one-dimensional nanowires that are either metallic or semiconducting, and they readily accept charges which can be transported along the tubular SWNT axis. The carbon fiber microelectrode (CFME) has been used in electrochemistry and in biological research fields because of its unique characteristics. Modifications to the CFME surface or carbon nanoelectrode have been used to produce sensing electrodes for some interesting biomolecules [3-7]. Significant efforts have been made for the application of CNTs due to their excellent biocompatibility and electron transfer ability. Recently CNTs have been intensively employed as modification materials in the surface of glassy carbon, graphite, carbon fiber and platinum electrodes. The modified electrodes have been applied to the detection of many biomolecules, such as catecholamines containing dopamine (DA) [8-15], indolealkylamines containing serotonin (5-HT) [14, 15], glucose [16-18], dihydronicotinamide adenine dinucleotide (NADH) [19-21], hydrogen peroxide [22-24], amino acids [25], AA [26] and heavy metals [27].

A few studies have reported research on the use of carbon fiber micro- or nanoelectrodes. Carbon fiber nanoelectrodes modified by SWNTs in sodium dodecylsulfate surfactant solution showed high sensitivity to DA, epinephrine (EP) and norepinephrine (NE) [4]. The use of MWNTs and Nafion to modify CFME greatly increased the sensitivity and selectivity of DA detection over AA, with a detection limit of 70 nM [5], suggesting further studies on disk form CFME modified by SWNTs and Nafion. Therefore, this study was conducted to demonstrate the enhanced response of the Nafion-SWNTs/CFME modified microelectrode toward DA in the presence of AA. The prepared microelectrode comprised SWNTs with a high surface area and a small amount of Nafion as the surfactant and negatively charged polymer [28-31]. The electrochemical determination of DA was performed without any interference from the AA contained in the solutions, and the modified microelectrode provided clear advantages such as excellent reproducibility and stability, rapid response, and a very low detection limit of 5 nM.

## 2. Results and Discussion

### 2.1 Electrochemical properties of dopamine at the Nafion-SWNTs/CFME modified microelectrode

**Figure 1.**
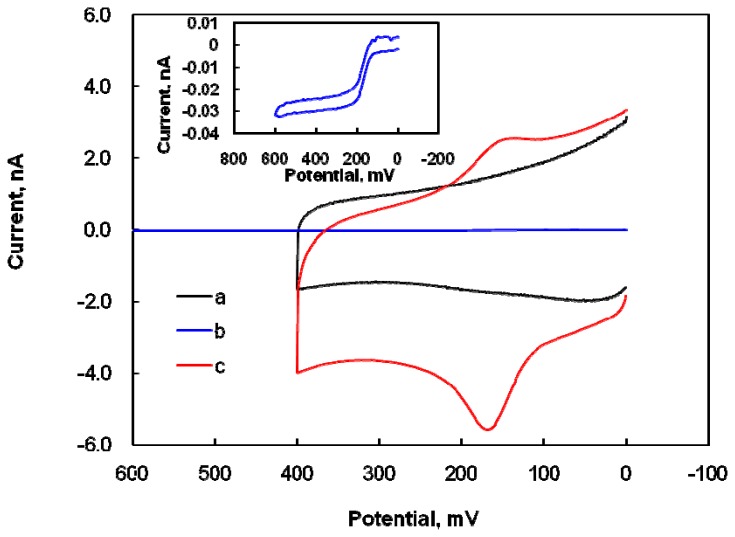
Cyclic voltammograms of a blank PBS solution: (a) at the modified microelectrode; (b) for the electrochemical response of 10 μM DA at a bare CFME and (c), at the modified microelectrode in PBS solutions at pH 7.4 with a scan rate of 10 mV/s. Inset: enlargement of (b).

The composite of SWNTs and Nafion in the electrode film should promote the selectivity and sensitivity of DA detection, despite the presence of the interfering molecule AA, because SWNTs have a high surface area and Nafion is a negatively charged polymer and surfactant. The electrochemical behaviors of the DA, at the modified microelectrode in 0.1 M PBS solutions (pH 7.4) were studied by CV. The cyclic voltammograms of 10 μM DA is shown in Figure 1.

There was no voltammetric response at the modified microelectrode in the blank PBS solution (Figure 1a). In Figure 1b, the representative cyclic voltammogram with a sigmoid shape at bare CFME (see inset: enlargement of Figure 1b) indicates that the electrochemical response of the bare CFME to DA is very poor. After modification with a suitable amount of Nafion-SWNTs, a well-defined, peak-shaped, redox couple appeared at an anodic peak potential of 0.18 V and a cathodic peak potential of 0.15 V in the 10 μM DA solution, as shown in Figure 1c. This tremendous enhancement in the anodic peak current of DA oxidation and the slightly negative-shift of overpotential confirm the electrocatalytic effects of the modified microelectrode on DA. These effects were attributed to the larger available surface area of the modified electrode, due to the nanometer dimensions of nanotubes, and the excellent electrical characteristics of the Nafion-SWNTs film. When compared with that for bare CFME (Figure 1b), the modified microelectrode (Figure 1c) greatly increased the peak current and the quasi-reversible electron transfer for DA, while the shape of the voltammograms was changed from sigmoid to peak-shaped at the modified electrode. However, the background current at the modified microelectrode (Figure 1c) was also much larger than that at bare CFME (Figure 1b), indicating that the Nafion-SWNTs/CFME modified electrode has a greater surface area and better conductivity. The peak separation (30 mV) between the oxidation and reduction peak potentials implied that two electrons are involved in the electrochemical process of DA, which is a comparable result to a previous report that DA is oxidized to DA *o*-quinone by the process of two electrons and two protons [32]. On the reversal cathodic scan, one reduced wave was obtained from the reduction of DA *o*-quinone.

**Figure 2.**
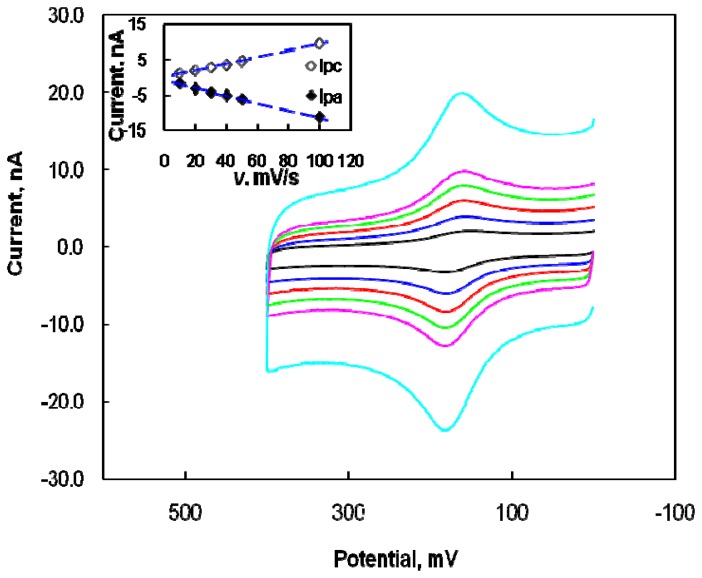
Cyclic voltammograms obtained at different scan rates from the modified microelectrode in a PBS solution at pH 7.4 containing 10 μM DA. Scan rates: 10, 20, 30, 40, 50, and 100 mV/s. Inset: plots of anodic and cathodic peak currents as a function of scan rate.

Figure 2 illustrates the effect of scan rate on DA oxidation at the modified microelectrode by CV. The anodic peak current of the modified electrode in the DA solution increased linearly with the scan rate, confirming the direct electron transfer on the surface of the modified electrode between it and DA.

Figure 2 (inset) presents the reasonable linearity of the plots, with correlation coefficients of 0.999 for cathodic current and 0.997 for anodic current. Therefore, the electrode reaction of DA at the modified microelectrode seems to be an adsorption-controlled process. The effect of accumulation time in the 10 μM DA solution on the anodic peak current was studied at the modified microelectrode. The anodic peak current of DA was almost constant after 30 sec, suggesting that an accumulation time of 30 sec is adequate to obtain the saturated current of the modified electrode and this time was used in subsequent experiments. As DA (pK_a_=8.87) is positive charged in a 0.1 M PBS solution at pH 7.4, it should be attracted to the negatively charged Nafion with sulfate functional groups by electrostatic interaction. The reproducibility and stability of the modified microelectrode were investigated. The modified microelectrode was cleaned by immersion into a blank PBS solution with stirring, after which each voltammetric measurement of DA in 0.1 M PBS solution was conducted. A voltammetric response was recorded in the blank PBS solution beforehand, and then the electrochemical response to DA was recorded in the DA solution. The result indicated that the anodic current was almost identical after 50 continuous potential scans, and that the voltammetric response after 30 days was also almost the same as that of the first scan when the modified electrode was maintained in air. Because the oxidation current of DA was very increased in 0.1 M PBS solution, DA determination was investigated in the presence of AA as an interfering molecule.

### 2.2 Determination of DA at the Nafion-SWNTs/CFME modified microelectrode

**Figure 3.**
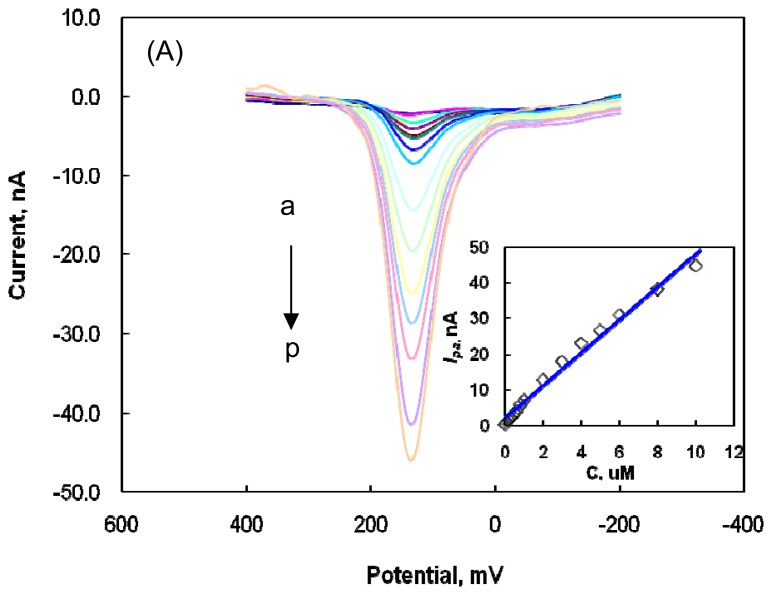

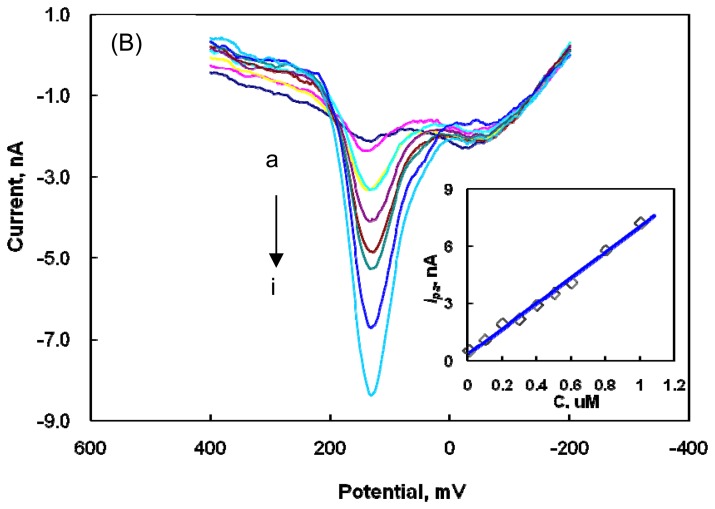
(A) DPV voltammograms with the subtraction of background current for 0.01, 0.1, 0.2, 0.3, 0.4, 0.5, 0.6, 0.8, 1.0, 2.0, 3.0, 4.0, 5.0, 6.0, 8.0, and 10 μM DA (a-p, respectively) at the modified microelectrode in a PBS solution at pH 7.4 with a 10 mV/s scan rate, 50 mV pulse amplitude, and 50 msec pulse width. Inset: plot of oxidative current vs. DA concentration (2.0-10 μM). (B) Enlargement of DPV voltammograms obtained from 0.01, 0.1, 0.2, 0.3, 0.4, 0.5, 0.6, 0.8, and 1.0 μM DA (a-i, respectively). Inset: plot of oxidative current vs. DA concentration.

Figure 3 illustrates a series of DPV voltammograms obtained for DA at different concentrations (10 nM - 10 μM) at the modified microelectrode in a 0.1 M PBS solution at pH 7.4.

The anodic peak current corresponding to the oxidation of DA was positively correlated with DA concentration (see inset of Figure 3). The linear regression equation was I_pa_/nA = 3.946 [C]/ μM + 6.496 in the concentration region of 2 μM-10 μM (Figure 3A), with a correlation coefficient of 0.993 and a DA detection limit of 17 nM. In the concentration region of 10 nM-1 μM (Figure 3B), the linear regression equation was I_pa_/nA = 6.672 [C]/ μM + 0.370, with a correlation coefficient of 0.994. The detection limit was 5 nM at a signal-to-noise ratio (S/N) of 3 in PBS solution at pH 7.4, representing a biological pH. The relative standard deviation of the same modified microelectrode in five successive scans was about 2 % for 1 μM DA, confirming the significant reproducibility of the modified microelectrode.

**Figure 4.**
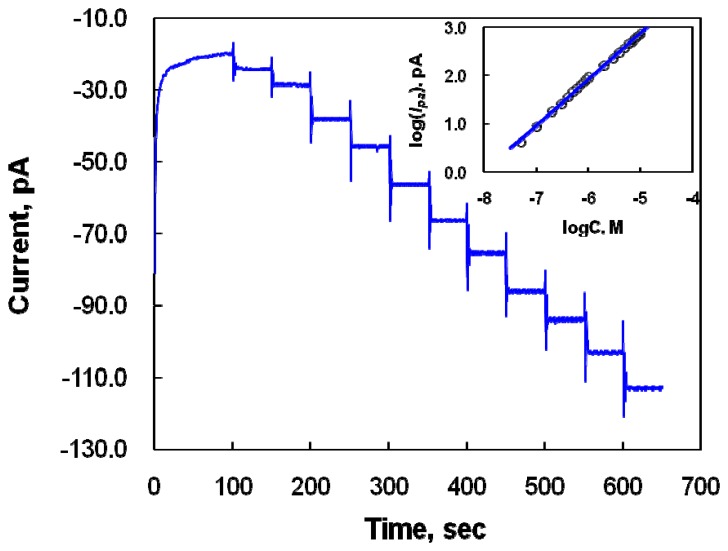
Amperometric response of DA concentrations of 0.05, 0.1, 0.2, 0.3, 0.4, 0.5, 0.6, 0.7, 0.8, 0.9, and 1.0 μM (a-k, respectively) at the modified microelectrode in a PBS solution at pH 7.4. The current was measured at a constant potential of 0.2 V corresponding to the DA oxidation potential. Inset: calibration plot (logI vs. logC_DA_) for DA obtained from amperometric measurements.

Figure 4 illustrates the amperometric responses of the modified microelectrode at 0.2 V to successive additions of DA (50 nM to 10 μM) to the 0.1 M PBS solution. The applied potential of 0.2 V was related to the oxidation potential of DA. The response time was usually less than 5s, depending on the DA concentration, confirming the fast amperometric response of the modified microelectrode to DA. The calibration plot (logI vs. logC_DA_) for DA obtained from the amperometric measurements is shown in Figure 4 (inset). The logarithm of the oxidative currents increased linearly with the logarithm of the DA concentrations in the range of concentration from 50 nM to 10 μM, with the linear regression equation of logI/pA = 0.953logC_DA_/M + 7.646 and a correlation coefficient of 0.998. DA concentrations can simply be determined *in vivo* using this modified electrode. The voltammetric response at the modified microelectrode was about 100-fold more sensitive than that at bare CFME.

### 2.3 Determination of DA in the presence of AA at the Nafion-SWNTs/CFME modified microelectrode

**Figure 5.**
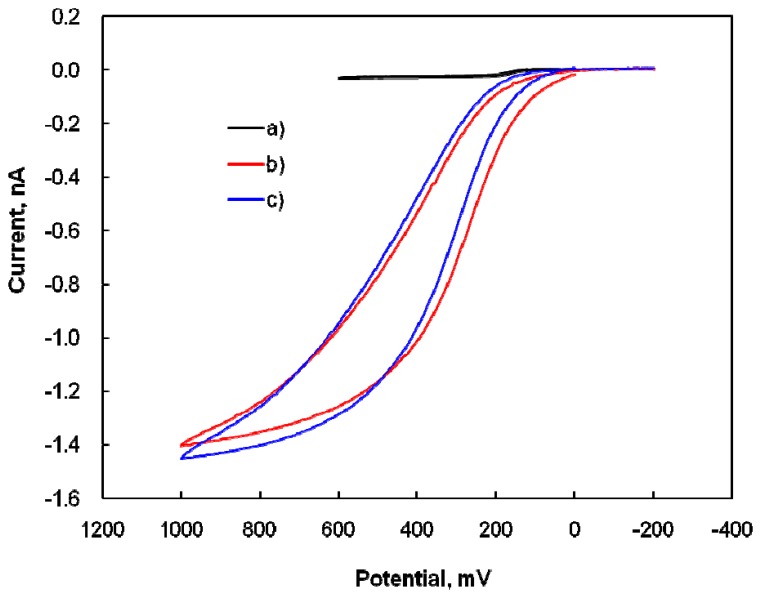
Cyclic voltammograms for the electrochemical response of 10 μM DA (a), 1.0 mM AA (b), and the mixture of 10 μM DA and 1.0 mM AA (c), at a bare CFME with a scan rate of 10 mV/s.

DA has become recognized as an important biomolecule that has received considerable attention due to its relation with Parkinson disease, and the selective determination of DA in the presence of various interfering molecules is a very important topic in the field of Analytical Chemistry. DA determination in the presence of AA was investigated in order to establish an acceptable level of selectivity. Figure 5 shows the CV curves obtained at a bare CFME in 10 μM DA, 1 mM AA, and their solution mixture. The anodic waves of DA and AA overlapped completely when all CVs were considered, and it was very difficult to distinguish between the anodic current of DA in the presence of AA at a bare CFME. Therefore, the possibility of detecting DA in the presence of AA was tested by the modified microelectrode. First of all, because Nafion, comprising a negatively charged polymer, was used in the preparation of the Nafion-SWNTs/CFME modified microelectrode, the electrochemical response of AA induced by the negative charge in the 0.1 M PBS solution was eliminated by the electron repulsion between Nafion and AA. OSWV was used for better voltammogram resolution.

**Figure 6.**
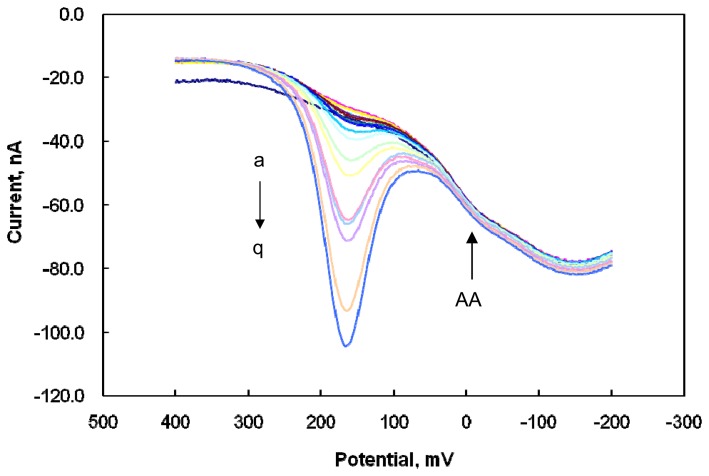
OSWV voltammograms of 1.0 mM AA in the presence of concentrations of DA of 0.0, 0.01, 0.1, 0.2, 0.3, 0.4, 0.5, 0.6, 0.8, 1.0 2.0, 3.0, 4.0, 5.0, 6.0, 8.0, and 10 μM DA (a-q, respectively) at the modified microelectrode in a PBS solution at pH 7.4. Inset: plot of oxidative current vs. DA concentration in the constant AA concentration of 1.0 mM with 2 mV step potential, 25 mV amplitude, and 10 Hz frequency.

Figure 6 shows the voltammograms of AA and DA with successive additions of DA at a constant AA concentration at the modified microelectrode. The anodic peak potential (-0.01 V) of AA was shifted negatively compared with that of the bare electrode, and its current was very poor, even at the high AA concentration of 1 mM. The DA oxidation currents were increased with increasing DA concentration, but the small anodic current of 1 mM AA was constant with successive DA additions. The obtained voltammograms show that the presence of AA exerted no effect on DA determination, suggesting that the DA concentration was detectable without any interference from AA, even at excessive AA concentration. The anodic peak potentials of both DA and AA at the modified microelectrode were shifted to more negative values compared with those at the bare CFME, with a larger shift of the AA potential, confirming the superior electrocatalytic oxidative characteristics of the modified microelectrode to both DA and AA. It can be concluded that the negatively charged polymer of Nafion was used for the DA attraction via electrostatic interaction and for the AA exclusion via electrostatic repulsion in the modified film on the CFME surface, and that SWNTs greatly promoted the electrocatalytic oxidation to DA due to the high surface area and electrical properties of SWNTs. The amount of Nafion in the modified film is therefore very significant for the voltammetric responses of DA and AA, and the ratio of Nafion/SWNTs was optimized for effective and selective DA detection in the presence of AA. Increasing ratio deteriorated the DA detection, whereas decreasing ratio increased the AA detection, so the optimal ratio of Nafion/SWNTs (w/w) for effective DA detection in the presence of AA was determined to be 0.1.

## 3. Experimental Section

### 3.1 Reagents and electrochemical apparatus

SWNTs (1.2–1.5 nm in diameter produced by the arc method) purchased from Aldrich were purified with 5.0 M HCl solution. Nafion (5 wt.% in lower aliphatic alcohols and water) DA, and L-AA were purchased from Aldrich. All other reagents used were of analytical grade. The phosphate buffer saline (PBS) solution was adjusted to a pH of 7.4 with 0.1 M H_3_PO_4_ and 0.1 M NaOH. High purity argon was used for deaeration. All experiments were carried out at room temperature (20 °C). Doubly distilled water with resistibility over 18 MΩ cm in a quartz apparatus was used to prepare all aqueous electrolyte solutions. Cyclic voltammetry (CV), differential pulse voltammetry (DPV), and Osteryoung square wave voltammetry (OSWV) were accomplished with a three-electrode potentiostat [Bioanalytical Systems (BAS) 100B/W] in a ground Faraday cage. DPV conditions were 10 mV/s scan rate, 50 mV pulse amplitude, 50 msec pulse width, and 30 s quiet time for DA determination in the presence of AA. OSWV conditions were 2 mV step potential, 25 mV amplitude, 10 Hz frequency and 30 s quiet time for DA determination in the presence of AA. A platinum-wire electrode was used as an auxiliary electrode. A Ag/AgCl electrode supplied by BAS was used as the reference electrode. CFME (disk electrode form – 11 μm in diameter; was purchased from BAS. The Nafion-SWNTs/CFME modified microelectrode was used as the working electrode. All potentials were reported with respect to the Ag/AgCl electrode at room temperature under argon atmosphere. The pH measurements were performed by pH glass electrode with a JENCO meter.

### 3.2 Preparation of the Nafion-SWCTs film based on CFME

The CFME surface was highly polished with alumina paste, sonicated with ultrasonic agitation, washed with 1.0 M HCl solution, and then rinsed several times with distilled water and methanol. After being cleaned, the CFME was immersed into the dispersed solution for 5 min, and then dried carefully under room temperature. The Nafion-SWNTs/CFME modified microelectrode was prepared for use as a working electrode. The composition of the dispersed solution was 1 mg SWNT and 0.01 % Nafion in 1 mL ethanol/water, and for the homogeneous suspension the solution was sonicated with ultrasonic agitation for 30 min.

## 4. Conclusions

The results confirmed that the Nafion-SWNTs modified disk form CFME possesses the obvious advantage of easy preparation in a rapid and simple procedure, effective electrocatalytic properties, and very low detection limits for DA, even in the presence of a 100 to 1000-fold excess of AA. Meanwhile, the results obtained from each CV, DPV, and OSWV methods for DA determination were comparable. The modified microelectrode demonstrated tremendous enhancements of sensitivity and selectivity. The combination of the good electrical properties of SWNTs with the negatively charged polymer of Nafion on the CFME surface has realized an efficient electrochemical microsensor capable of maintaining reproducibility and stability. The resulting technique can be used to monitor DA concentrations at nM levels in microvolume samples.
