# Jeong, H.; Jeon, S. Determination of Dopamine in the Presence of Ascorbic Acid by Nafion and Single-Walled Carbon Nanotube Film Modified on Carbon Fiber Microelectrode. *Sensors* 2008, *8*, 6924-6935

**DOI:** 10.3390/s90100376

**Published:** 2009-01-08

**Authors:** Haesang Jeong, Seungwon Jeon

**Affiliations:** Department of Chemistry and Institute of Basic Science, Chonnam National University, Gwangju, 500-757, Korea

By mistake, we omitted the support from Chonnam National University in the Acknowledgements section in our paper recently published in *Sensors* [1]. Therefore, the Acknowledgements section is revised as follows:
